# The Efficiency of Atmospheric Dielectric Barrier Discharge Plasma against *Escherichia coli* and *Bacillus cereus* on Dried Laver (*Porphyra tenera*)

**DOI:** 10.3390/foods9081013

**Published:** 2020-07-28

**Authors:** Ji Yoon Kim, Eun Bi Jeon, Man-Seok Choi, Eun Ha Choi, Jun Sup Lim, Jinsung Choi, Shin Young Park

**Affiliations:** 1 Institute of Marine Industry, Gyeongsang National University, Tongyeong 53064, Korea; yooonn22@naver.com (J.Y.K.); eunb61@naver.com (E.B.J.); pyn358@naver.com (M.-S.C.); 2 Department of Seafood and Aquaculture Science, Gyeongsang National University, Tongyeong 53064, Korea; 3 Department of Electrical and Biological Physics, Plasma Bioscience Research Center, Kwangwoon University, Seoul 01987, Korea; ehchoi@kw.ac.kr (E.H.C.); junsub117@gmail.com (J.S.L.); neoled@kw.ac.kr (J.C.)

**Keywords:** dried laver, DBD plasma, *Escherichia coli*, *Bacillus cereus*, quality

## Abstract

This study investigated the effects of atmospheric dielectric barrier discharge (DBD) plasma (1.1 kV, 43 kHz, 5–30 min, N_2_: 1.5 L/m) on the reduction of *Escherichia coli* and *Bacillus cereus* on dried laver. The reductions of *E. coli* and *B. cereus* by 5, 10, 20, and 30 min of DBD plasma were 0.56 and 0.24, 0.61 and 0.66, 0.76 and 1.24, and 1.02 and 1.38 log CFU/g, respectively. The D-value of *E. coli* and *B. cereus* was predicted as 29.80 and 20.53 min, respectively, using the Weibull model for *E. coli* (R^2^ = 0.95) and first-order kinetics for *B. cereus* (R^2^ = 0.94). After DBD plasma 5–30 min treatment, there was no change in pH (6.20–6.21) and this value was higher than the untreated dried laver (6.08). All sensory scores in DBD plasma-treated laver were determined as >6 points. The 30 min of DBD plasma is regarded as a novel intervention for the control of potential hazardous bacteria in dried laver.

## 1. Introduction

Laver (*Porphyra tenera*) belongs to Rhodophyta, Bangiophyceae, Bangiales. It is considered a traditional and popular seaweed food in Korea and Japan [1]. With the development of various dried seaweed products, the area of consumption has expanded not only for side dishes, but also for snacks with alcoholic beverages, and it has now become an indispensable part of the Korean meal. Dried laver is a representative seaweed produced in Korea and is produced in the order of seaweed and kelp, with production of 210,000 tons, accounting for about 27 percent of total algae production [2]. The locally produced seaweed is mainly exported to Japan, the United States, Taiwan, and Thailand, following tuna as the leading export item for fishery products. As exports gradually increase, concerns about the hygiene of dried laver are also growing. Since seafoods such as laver, kelp, and sea mustard are produced in seawater environment inhabited by various microbial species, they naturally contain high numbers of diverse microbial populations; they are also known to be active in microbial growth and metabolism if exposed to the outdoor environment for a long time during production and distribution [3]. Dried laver is very likely to be contaminated by microorganisms during the manufacturing process. In the case of laver, it is likely to be contaminated by sea water, air, the drying process, and secondary contamination, such as by hands, and is reported to contain about 1.0 × 10^6^ CFU/g of total cell count [1,4]. According to Lee et al. [1], 1.5 × 10^2^ CFU/g of *E. coli* and 2.1 × 10^2^ of *B. cereus* CFU/g were detected in dried laver distributed in commercial markets. Although the importance and understanding of laver hygiene has been increasing, research on the microbial reduction treatment of dried laver is still in incomplete initial stages [5].

Plasma, as partially ionized gas, is the state of the fourth substance containing ions, electrons, atoms, ions, and reactive neutral species. Plasma is receiving attention for its use in microbial sterilization technology, which allows both physical and chemical treatment, because it includes active radicals, ultraviolet rays, and infrared rays that are highly reactive in chemicals, as well as charged particles [6]. An atmospheric dielectric barrier discharge (DBD) device consists of two metal electrodes and a low temperature plasma device, which limits electric current by covering one or two metal electrodes. The purpose is to discharge atmospheric pressure by placing a voltage between the two electrodes [7]. Among these devices, atmospheric pressure plasma technology, which can produce plasma at low temperatures under atmospheric pressure, is being studied for application in food and medical life sectors. Atmospheric DBD plasma is regarded as a promising next-generation non-thermal technology for food sterilization. As DBD plasma device generates plasma under atmospheric conditions, this operation is simple and continuous. Therefore, atmospheric DBD plasma can be applied in agriculture and food industries at a low cost. Despite these advantages, it has not been systematic, extensive and deeply conducted in Korea. Therefore, the application of the industry field is not being carried out quickly [8]. Some studies have demonstrated that DBD plasma is effective against pathogenic microorganisms—*Escherichia coli*, *Listeria*, human norovirus, and *Neurospora crassa* [6,9,10,11]. Furthermore, DBD plasma, which can be used instead of chemical preservatives, was specifically used against *Staphylococcus aureus* and *E. coli* for dried seaweed products and dried fishery food products. This plasma can be used as a very suitable food preservation method [12].

There is a need to further investigate various methods for the reduction of microorganisms and the effects of atmospheric DBD plasma on food decontamination without affecting overall quality. The objective of our investigation was to study the effectiveness of non-thermal atmospheric DBD plasma (1.1 kV, 43 kHz, N_2_: 1.5 L/m) for 5–30 min in combating *E. coli* and *B. cereus* on dried laver, which could be one of the potent carriers of foodborne bacteria. This study was also aimed at finding out whether the DBD plasma treatment had an adverse effect on chemical property (pH) and sensory attributes of dried laver.

## 2. Materials and Methods

### 2.1. Bacterial Strain

*Escherichia coli* (ATCC 11,229, KCCM 11,234, and KCCM 12,181) and *Bacillus cereus* (NCCP 10,623, NCCP 14,579, and ATCC 1178) stock cultures were stored at −80 °C in trypsin soy broth (TSB, Difco Laboratories, Detroit, MI). Each bacteria was cultivated in tryptic soy broth (TSB, Difco Laboratories, Detroit, MI, USA) at 37 °C for 24 h and this process was repeated twice for bacterial activation.

Each strain was incubated at 37 °C for 24 h in 5 mL TSB and centrifugation was performed at 5400 rpm for 10 min at 4 °C. The pellets obtained by centrifugation were resuspended in 9 mL of 0.85% sterilized NaCl solution and *E. coli* and *B. cereus* were mixed with each of the three pellets.

### 2.2. Inoculation of Dried Laver

The dried laver was purchased in a market in Korea. The dried laver was cut into 30 × 30 (L × W) mm pieces with sterilized scissors. The surface of the dried laver was disinfected with 70% ethanol to remove natural microorganisms and dried in a biological safety cabinet (CHC Lab Co. Ltd., Daejeon, Korea). Each cut and sterilized piece was used as a sample in this study.

Next, 0.5 g of the cut and dried laver was put into petri dishes (60 × 15 mm) for inoculation. The final concentrations of activated *E. coli* and *B. cereus* were approximately 8–9 and 7–8 log_10_ CFU/g, respectively. Each bacterial strain was widely spot-inoculated at 100 µm on the front surface of the laver. The inoculated sample was immediately dried in the biological safety cabinet.

### 2.3. Atmospheric DBD Plasma Treatment

**Figure 1:**
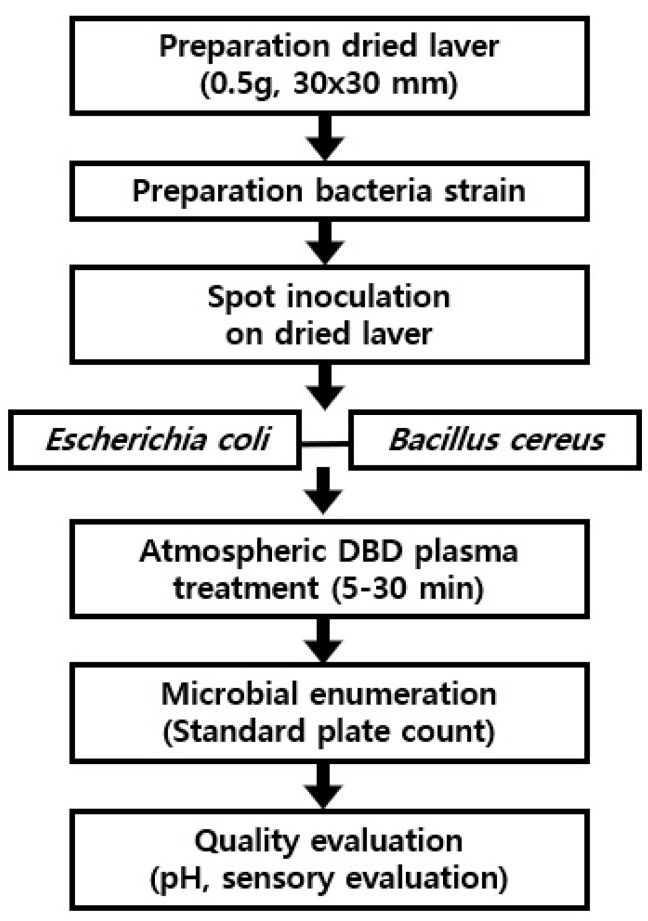
A flow diagram for atmospheric dielectric barrier discharge (DBD) plasma treatment of dried laver.

**Figure 2:**
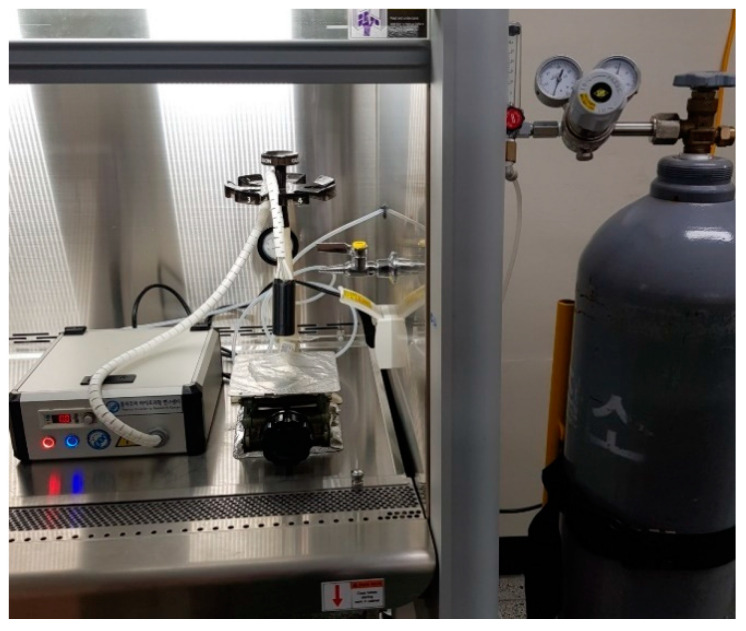
Atmospheric dielectric barrier discharge (DBD) plasma device used in experiment (1.1 kV, 43 kHz, N_2_: 1.5 L/m).

The overall process for atmospheric DBD plasma treatment is shown in Figure 1. For atmospheric DBD treatment, 0.5 g of dried laver was put into a petri dish. Samples were treated at different times, respectively (5–30 min). The dielectric barrier discharge (DBD) plasma device (μ-DBD Surface Plasma Generator, Model; Micro DBD plasma) is shown in Figure 2 and was supplied by Plasma Biomedicine Institute (Plasma Bioscience Research Center, Seoul, Korea) and was reported by Ryu et al. [6]. The silver electrode that served as a high voltage electrode was screen printed (thickness of 10 µm) on glass (thickness of 1.8 mm); the dielectric material, which consisted of SiO2, was also screen-printed at 100 µm in polylactic acid. A metal mesh grid was attached to the rear side of the glass and used as a grounded electrode. Gas could flow toward the mesh surface by polylactic acid through a gas injection hole. DBD plasma was generated on the rear glass surface between the glass and metal mesh grid using a nitrogen flow rate of 1.5 L/min. The DBD plasma under a driving frequency of 43 kHz showed voltage and current characteristics with low discharge voltage of approximately 1 kV and discharge peak current of 40 mA, respectively. The minimum discharge voltage for plasma production by DBD plasma devices used in this experiment was 1.1 kV. The optical emission profile was measured at a distance of 3 mm from the mesh surface with 10 sec integration time using a spectrometer (HR4000, Ocean Optics). The device was turned on at least 10 min before the start of the experiment and the surface of the dried laver inoculated with *E. coli* or *B. cereus* was treated with DBD plasma for 5, 10, 20, and 30 min in a sterile petri dish (35 × 15 mm). A distance of 3 mm was kept between the plasma-emitting electrode and the sample during treatment.

### 2.4. Microbial Enumeration

For enumeration of *E. coli* and *B. cereus* after the plasma is treated, the samples are put into a sterilized bag (Labplus Inc., Sainte-Julie, Quebec, Canada) with 0.85% sterile NaCl solution and homogenized in a stomacher (Easy Mix, AES Chemunex, Rennes, France). The homogenized specimen solution was diluted with 9 mL of 0.85% sterile NaCl solution in steps. One mL of diluted samples was poured on the tryptic soy agar (TSA, Difco Laboratories, Detroit, MI, USA) plate for *E. coli* or *B. cereus*. The samples were incubated in an incubator at 37 °C for 24 h. Fifteen to 300 colonies per plate were calculated by selecting the plates, and the unit of microbial colonies was indicated by Colony Forming Unit (CFU/mL) per 1 mL of the specimen solution.

### 2.5. Determination of D-Values

Two models were used for each microorganism to measure the D-value of *E. coli* and *B. cereus.* The Weibull model, a two-parameter nonlinear model, was used for the D-value calculation of *E. coli* using the following expressions:

(1) $$\mathrm{Log}\left(\frac{N_{t}}{N_{0}}\right)=-bt^{n}$$

where N_t_ is the population of microorganism (log CFU/g) after an exposure time *t*, N_0_ is the initial population of microorganism (log CFU/g), *t* is the exposure time, *b* and *n* are the scale (a characteristic time). The *b* value represents the time needed to reduce the population for one log unit, while the *n* parameter indicates the shape of the survival curve. The *n* value of 1 corresponds to a linear survival curve, while *n* values correspond to downward and upward concavity, respectively.

For the calculation of D-value from the Weibull parameters, an equation was used as by Buzrul and Alpas [13]:

(2) $$D={\left(\frac{1}{b}\right)}^{1/n}$$

where D indicates the time required to reduce a microorganism by 90%. The Weibull model was fitted by nonlinear regression, using the software GraphPad Prism version 5.0 for Windows (GraphPad Software, San Diego, CA, USA).

The first-order kinetics model was used to calculate the reduction in *B. cereus* of dried laver.

(3) $$\log\frac{N_{0}}{N}=\frac{k}{2.303}\cdot t$$

where:

▪ N_0_ is the initial microbial population (CFU/g)

▪ N is the microbial population by DBD treatment time (CFU/g)

▪ t is the exposure time (min)

▪ k is the reduction rate constant

It can be characterized by a single rate constant “k” or its reciprocal, the D-value, which provides a quantitative measure of resistance to an applied lethal agent. D-value refers to decimal reduction time.

### 2.6. pH Value Analysis

For the measure of pH value, 1 g of dried laver was mixed with 9 mL of diluted water and stirred for 5 min at room temperature for use. The pH value was measured three times repeatedly using a pH meter (Orion Star A211, Thermo Scientific, MI, USA).

### 2.7. Sensory Evaluation

For sensory evaluation, the dry laver that treated the plasma was provided to the panel. The panel consisted of men and women in their twenties, college and graduate students (male: 15, female: 15) of the Department of Seafood and Aquaculture Science at Gyeongsang National University.

The samples were evaluated for color, flavor, texture, appearance, and overall acceptability. The quality was assessed using the 7-point scale, represented as follows: “1”—extreme dislike, not acceptable; “4”—neither like nor dislike, the lower limit of the acceptable range; and “7”—extreme like, essentially free from any defect, original quality preserved. A rating higher than 4 indicated greater acceptability of the food product. Prior to sample evaluation, trained panelists participated in orientation sessions to familiarize themselves with the scale attributes of the hedonic scale and the related quality assessment. The panelists evaluated the samples independently in identical environments.

### 2.8. Statistical Analysis

Statistical processing of the analysis results was carried out as three iterations per specimen for all experiments. The statistics program used one-way ANOVA and Duncan’s multi-range test in the SPSS version 12.0 software program (SPSS Inc., Chicago, IL, USA) and verified the significant difference at the level of 5% (*p* < 0.05).

## 3. Results

### 3.1. Effects of Atmospheric DBD Plasma on the Reduction of E. coli and B. cereus in Dried Laver

**Figure 3:**
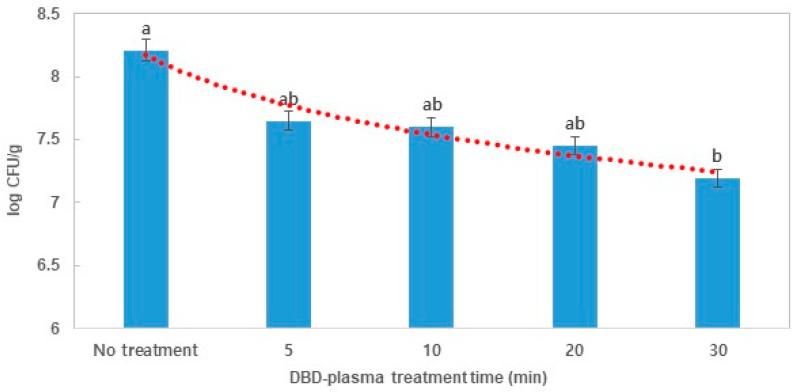
Effects of DBD-plasma treatment on *E. coli* in dried laver and fitted survival curves by the Weibull model. The letters a and b indicate significant differences (*p* < 0.05) in reduction over DBD plasma treatment time for *E. coli* (Duncan’s multiple range test with 5% probability).

**Figure 4:**
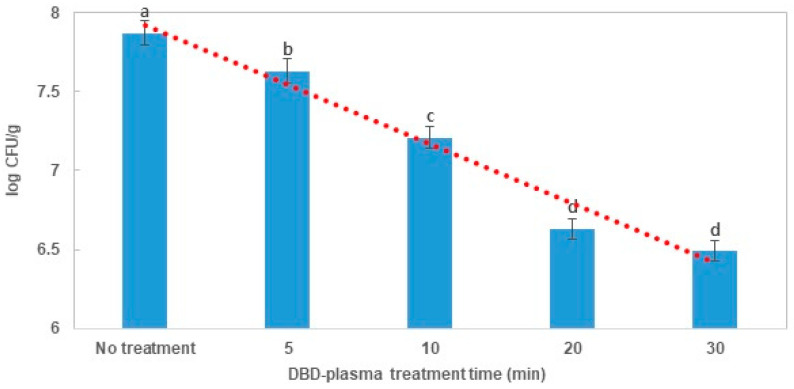
Effects of DBD-plasma treatment on *B. cereus* in dried laver and fitted survival curves by the first-order kinetics. The letters a, b, c, and d indicate significant differences (*p* < 0.05) in reduction over DBD plasma treatment time for *B. cereus* (Duncan’s multiple range test with 5% probability).

To evaluate the bactericidal effects of DBD plasma on *E. coli* and *B. cereus* in dried laver, the overall average populations of *E. coli* and *B. cereus* for dried laver are shown in Figure 3 and Figure 4. The populations of *E. coli* and *B. cereus* gradually decreased with the increase in treatment time (5–30 min) on the laver with DBD plasma. There were no significant differences (*p* > 0.05) in the populations of *E. coli* with 5, 10, and 20 min of DBD plasma treatment; the overall average counts of *E. coli* in the laver treated for 5, 10, and 20 min with DBD plasma were 7.65 (reduction of 0.56 log, 72.46%), 7.60 (reduction of 0.61 log, 75.45%) and 7.45 (reduction of 0.76 log, 82.62%) CFU/g, respectively. However, there was a significant decrease (*p* > 0.05) in *E. coli* in the laver treated for 30 min with DBD plasma that showed 7.19 log CFU/g (reduction 1.02 log, 90.45%) when compared with the initial *E. coli* count of 8.21 log CFU/g (Figure 3). Survival curves of *E. coli* (Figure 3) by DBD plasma treatment were fitted using the Weibull model, which is used for nonlinear microbial survival. The parameters (b, *n*, D, R^2^) of the Weibull model are shown in Table 1. R^2^ of *E. coli* was measured at 0.95, and D-value was 29.80 min. The goodness fit of the model was estimated by R^2^ and since all of the values were close to 1.0, this confirmed that the model was a good fit to the survival curves.

*B. cereus* counts were significantly (*p* < 0.05) reduced in the dried laver by increasing DBD plasma treatment time (5–30 min) (Figure 4). However, no significant differences (*p* > 0.05) in *B. cereus* were observed between the laver treated with DBD plasma for 20 min and the laver treated with DBD plasma for 30 min. The overall average counts of *B. cereus* with 5, 10, 20, and 30 min of DBD plasma were 7.63 (reduction of 0.24 log, 42.46%), 7.21 (reduction of 0.66 log, 78.12%), 6.63 (reduction of 1.24 log, 92.25%), and 6.49 (reduction of 1.38 log, 95.83%) CFU/g as compared with the initial *B. cereus* counts of 7.87 log CFU/g, respectively. Based on the *B. cereus* survival curves in the dried laver, the D-values were calculated using first-order kinetics (Table 1). The R^2^ values (0.94) indicated that this log-linear kinetic model for *B. cereus* was a suitable fit to determine the slopes and D-values.

The over 1 log reduction (90%) of the two main bacterial contaminants *E. coli* and *B. cereus* was only observed after the maximum treatment time with DBD plasma of 30 min. There were significant differences (*p* < 0.05) in D-values for the two bacteria in DBD plasma treated dried laver (Table 1).

### 3.2. pH and Sensory Evaluation

Table 2 shows changes in pH of dried laver by DBD plasma treatment time. The result of the control not treated with DBD plasma was 6.08. The results were 6.20 6.21, 6.21, and 6.21 for 5, 10, 20, and 30 min of DBD plasma treatment, respectively. There were significant differences between the control without plasma treatment and the samples with plasma treatment (*p* < 0.05), but no significant difference in the change in pH for the treatment at 5 and 30 min (*p* > 0.05).

The sensory evaluation items of dried laver included color, flavor, smell, and overall acceptability (Table 3). The results of dried laver’s overall acceptability showed that the control, which did not have DBD plasma treatment, was 6.83 points, followed by treatment for 5, 10, 20, and 30 min with 6.75, 6.58, 6.58, and 6.50 points, and no significant difference in all the groups (*p* > 0.05).

## 4. Discussion

The consumption of laver is gradually increasing due to the changes in dietary life that seeks health and convenience. Depending on the processing method, it is divided into various products, such as dried laver, grilled laver, and seasoned laver and is also distributed as a variety of products, such as snack laver with seasoning and fried laver with many different flavors and scents added [14]. Most of the laver was consumed domestically until the early 2000s, but exports have increased from $60 million in 2007 to about $1.5 billion in 2011, and $3.5 billion in 2016 [9], and topped the list of seafood products with $5.7 billion in 2019 [15]. In addition, the number of exporting countries more than doubled from 49 in 2007 to 109 in 2017 [16]. However, microbiological hazards management was insufficient in the production and processing stages and was focused only on increasing the production. Moreover, the age of consumption of laver varied from infants to the elderly and as a food with a high preference, safety issues are emerging [14]. However, only nine types of food poisoning bacteria are managed on the common basis of the general food standard according to whether it is produced without further processing or heating: *Salmonella* spp, *Staphylococcus aureus*, *Vibrio parahaemolyticus*, *Listeria monocytogenes*, *Escherichia coli* O157:H7, *Campylobacter jejuni*, *Yersinia enterocolitica*, and *Clostridium perfringens* are set to negative and *Bacillus cereus* must be less than 1000 CFU/g. The importance of laver hygiene is increasing and there are some studies on the microbial reduction of dried laver. However, there is still a lack of study on the non-thermal diverse microbial inhibition methods in dried laver. [5]. In particular, the hygienic problems of dried laver are mainly associated with foreign substances and microbial contamination. Foreign substances can be solved through rigorous and scientific inspection, but microbial contamination is caused by cross-contamination by raw materials, water, machine, and workers. The problem of contamination by raw materials and water is quite difficult to solve unless special measures are taken [17,18]. A study on dried laver treated with an electron beam [19] decreased initial total bacteria counts from 1.5 × 10^6^ to 5.4 × 10^4^ and 1.1 × 10^4^ with irradiation of 4 kGy and 7 kGy, respectively. A study on dried laver treated using intense pulsed light showed an apoptosis rate of 0.6 log CFU/g after 1 min of irradiation and 1.6 log CFU/g after 10 min [20].

Cold plasma such as DBD plasma emits antibacterial materials, including electrons, cations, anions, free radicals, neutral atoms, UV photons, reactive nitrogen species (RNS), and reactive oxygen species (ROS) [21,22]. When the target samples are exposed to the plasma, these particles have a sterilization effect by being in direct contact with the surface. According to Smet et al. [23], rupturing bacterial cell walls using an accumulation of charged particles or bombardment with free radicals have been proposed as possible means of action for bacteria inactivation. Moreover, ROS and RNS can attack both the cell envelope and components of microorganisms, causing cellular envelope collapse and damage to intracellular components (e.g., DNA). These active species can damage the cell lipid and the protein of the cell membrane, thus allowing for physical and chemical sterilization [24]. It was reported that the concentration of active species (ROS, RNS) can be proportionally increased as there is an increase in power and exposure time and exposure distance in DBD plasma treatment [6]. Treatment with atmospheric non-thermal dielectric barrier plasma (DBD) is receiving attention as a next-generation method of microbial control of agro-fishery products because it has less protein denaturation and quality change due to the heat. Although there is greater microbial reduction enabled by increasing the treatment power and time, in practice a possible negative effect on quality must be taken into account. Therefore, many studies on DBD plasma are actively underway, such as microbial decontamination of dried laver [25], the antibacterial activity of main food-borne bacteria [11], and microbial decontamination of vegetables and spices [9] using atmospheric DBD plasma treatment.

The study by Kim et al. [25] reported that DBD plasma treatment for 10 min reduced aerobic bacteria on dried laver by 2.5 log CFU/g. This result is a much higher reduction of aerobic bacteria when compared to the current results that showed DBD plasma for 10 min only reducing *E. coli* and *B. cereus* on dried laver by a 0.61–0.66 log-reduction. It can be considered that a much higher discharge voltage (10 kV) for plasma production was used in the study by Kim et al. [25] than in the current study (1.1 kV). Choi et al. [11] reported that the longer the DBD plasma treatment time was, the higher the sterilization effect for *S. aureus*, *B. cereus*, *V. parahaemolyticus*, *S*. *enterica* and *E. coli* in the suspension was. They also reported that 30 min of DBD plasma treatment resulted in 1-log reduction of *S. aureus* and *B. cereus* in dried blackmouth angler. *B. cereus* in sunsik (dried and powdered mixed vegetables) was reduced by 2.20 log CFU/g when treated with DBD plasma for 20 min [26]. When tiger nut milk was treated with DBD plasma for 8 min, aerobic bacteria and fungi were reduced to 0.97 and 0.94 log CFU/g, respectively [27]. Based on these studies, the microbial inhibitory efficacy could be greatly influenced by several factors such as microbial species, injected gas type, food matrix, and surface morphology of food.

A first-order kinetics model is traditionally used for determining inactivation data, such as D-values, and is based on linear microbial inactivation curves [28] and used for micro-decontamination of dried laver by DBD plasma treatment [25]. The Weibull model is also used for determining D-values and is based on nonlinear microbial inactivation curves [8]. Studies have suggested that the Weibull model likely provides a better fit than first-order models for kinetic analysis of non-thermal measures, such as study by Huang et al. [29], Park and Ha [30]. In this study, two models were examined to determine the best fit for describing the DBD plasma inactivation of *E. coli* and *B. cereus*. The Weibull model for *E. coli* and first-order kinetics for *B. cereus* were used to describe nonlinear and linear bacterial survival curves, respectively. The survival curves and D-values for *E. coli* in dried laver were obtained using the Weibull model. However, the Weibull model was not suitable for *B. cereus* inactivation and the data showed a better fit to a linear model rather than a nonlinear one. Therefore, a first-order kinetics model was used for the survival curves and D-values of *B. cereus*. In general, R^2^ values provided an indication of how closely the data fit to a first-order kinetics or Weibull model; the higher the R^2^ value (0 < R^2^ < 1), the better the model fit the data [31].

In this study, after DBD plasma treatment of dried laver, it was confirmed that there were no changes in overall quality that consumers sensed, although DBD plasma treatment of laver slightly increased its pH values in this study. A slight increase in pH values had no effect on the sensory aspects. This fact was similarly shown in a study by Bunz, Mese, Zhang, Piwowarczyk, and Ehrhardt [32].

## 5. Conclusions

This study was conducted to investigate the effects of DBD plasma on *E. coli* and *B. cereus* as the potential microbial hazards of dried laver. The reductions of *E. coli* and *B. cereus* in the laver treated by 5–30 min of DBD plasma were 0.56–1.02 and 0.24–1.38 log CFU/g, respectively. The D-value of *E. coli* and *B. cereus* was calculated as 29.80 and 20.53 min, respectively, using the Weibull model for *E. coli* (R^2^ = 0.95) and the first-order kinetics model for *B. cereus* (R^2^ = 0.94). No changes in pH and sensory scores (*p* < 0.05) were observed in the laver treated with DBD plasma for 5–30 min (pH 6.20–6.21, sensory scores >6 point). Thus, these findings suggest that 30 min of atmospheric DBD plasma treatment for dried laver can reduce the microorganisms without deleterious changes in food qualities. This study also provides the application of DBD plasma as a new emerging microbial control technology for the seaweed food industry.

## Figures and Tables

**Table 1:** Model parameters for *E. coli* and *B. cereus* of dried laver by DBD-plasma treatment.

Target Microorganism	Model Parameters		R^2^	D-Value ± SD (min)
*E. coli*	**b** ± SD	0.316 ± 0.059	0.95	29.80 ± 8.23 ^a^
	*n* ± SD	0.338 ± 0.064		
*B. cereus*	Decay slope	0.049 ± 0.003	0.94	20.53 ± 1.27 ^b^

The survival curves and D-values for *E. coli* were obtained using the Weibull model. The survival curves and D-values for *B. cereus* were obtained using the first-order kinetics model; **b**: scale parameter, concave upward survival curve if *n* < 1, concave downward if *n* > 1 and linear if *n* = 1; R^2^: correlation coefficient; Different superscript in a column (^a,b^) are significant differences (*p* < 0.05); Values mean±standard deviations of triplicate determination.

**Table 2:** pH value of the dried laver for the DBD plasma treatment.

Treatment Time (min)	pH
Control	6.08 ± 0.02 ^b^
5	6.20 ± 0.01 ^a^
10	6.21 ± 0.01 ^a^
20	6.21 ± 0.03 ^a^
30	6.21 ± 0.01 ^a^

Control is non-treatment by DBD plasma. Values are mean ± standard deviations of triplicate determination. Different superscript in a column ^a^ and ^b^ are significant differences (*p* < 0.05).

**Table 3:** Sensory evaluation of the dried laver for the DBD plasma treatment.

Treatment Time (min)	Sensory Evaluation			
	Color	Flavor	Smell	Overall Acceptability
**Control**	6.67 ± 0.52 ^a^	6.25 ± 0.42 ^a^	6.33 ± 0.52 ^a^	6.83 ± 0.41 ^a^
**5**	6.50 ± 0.55 ^a^	6.17 ± 0.41 ^a^	6.25 ± 0.42 ^a^	6.75 ± 0.42 ^a^
**10**	6.33 ± 0.52 ^a^	6.08 ± 0.20 ^a^	6.17 ± 0.75 ^a^	6.58 ± 0.49 ^a^
**20**	6.25 ± 0.42 ^a^	6.00 ± 0.55 ^a^	6.25 ± 0.42 ^a^	6.58 ± 0.49 ^a^
**30**	6.17 ± 0.41 ^a^	6.08 ± 0.20 ^a^	6.08 ± 0.20 ^a^	6.50 ± 0.45 ^a^

Control is non-treatment by DBD plasma. Values are mean ± standard deviations of triplicate determination. Different superscript in a column ^a^ and ^b^ are significant differences (*p* < 0.05).
